# The COVID-19 Vaccination Hesitancy Among the People With Inflammatory Bowel Disease in China: A Questionnaire Study

**DOI:** 10.3389/fpubh.2021.731578

**Published:** 2021-10-11

**Authors:** Xia Wu, Jue Lin, Heena Buch, Quchen Ding, Faming Zhang, Bota Cui, Guozhong Ji

**Affiliations:** ^1^Medical Center for Digestive Diseases, The Second Affiliated Hospital of Nanjing Medical University, Nanjing, China; ^2^Key Lab of Holistic Integrative Enterology, Nanjing Medical University, Nanjing, China

**Keywords:** inflammatory bowel disease, SARS-CoV-2 vaccination, perspective, willingness, immunocompromised population

## Abstract

**Objective:** To explore the attitudes and views of patients with inflammatory bowel disease (IBD) on COVID-19 vaccination.

**Methods:** An online interview questionnaire concerning the acceptance or hesitancy toward vaccination for COVID-19 was designed and 543 patients with IBD in China were invited to complete the structured self-administered anonymous questionnaire.

**Results:** Of all the participants, 50.7% were indecisive about the vaccination and only 16.0% opted for it. Vaccination hesitancy was significantly associated with women and those without medical or biomedical backgrounds. The acceptance of COVID-19 vaccination was higher in participants with no history of immune-modifying therapies, especially in those without immunosuppressants. Participants who considered vaccination critically important to self-health or the health of others were more likely to choose immediately or later vaccination. Safety and potential adverse reactions, personal hypoimmunity, efficacy, and reliability of COVID-19 vaccines were the top three concerns of the participants that were independent of their willingness for vaccination.

**Conclusions:** This study discloses the presence of hesitancy for COVID-19 vaccination in patients with IBD. Further studies are warranted to evaluate the efficacy and safety of COVID-19 vaccines in IBD individuals, with a specific focus on the impact of immune-modifying therapies. Health education and recommendation from authoritative sources may facilitate COVID-19 vaccination efforts.

## Introduction

The COVID-19 pandemic has claimed the lives of more than 4.45 million people worldwide (accessed August 26, 2021) (1), and led to substantial concerns for inflammatory bowel disease (IBD) patients due to the presence of dysbiosis and immunocompromised status, despite studies demonstrating similar risk and mortality between patients with IBD and the general population (2). The pandemic significantly affected the daily lives of patients with IBD compared with non-IBD patients (3). The fear of SARS-CoV-2 infection in patients with IBD is more pronounced, especially in those taking immunosuppressants (3, 4). IBD treatments involve many immunosuppressive medications controlling an overactive immune response to induce and maintain clinical remission (5). The long-term systemic corticosteroid (especially prednisone > 20 mg/day), sulfasalazine/mesalamine and thiopurine usage is associated with an increased risk of severe COVID-19, whereas tumor necrosis factor (TNF) antagonist therapy is not (6, 7). The management of IBD during the COVID-19 pandemic has been a challenge for clinicians and patients. Based on the suggestions of experts, IBD therapies other than prednisone should be continued to sustain remission in patients not infected with SARS-CoV-2 (5, 8). However, prednisone, thiopurines, methotrexate, and tofacitinib should be stopped if patients with IBD test positive for SARS-CoV-2 or develop COVID-19 (5, 8, 9). The use of biologics should be comprehensive, based on the severity of COVID-19 and IBD (5, 8).

Efforts have been made to prevent patients with IBD from COVID-19, including the management and treatment guidelines for patients with IBD (8, 9) and the development of related studies (10, 11). The development of safe and effective vaccines is the best way to mitigate against the risk of COVID-19 and prevent the situation from worsening. Despite the promising effectiveness and safety of the candidate vaccines, immunocompromised patients or patients on immunosuppressant medications were excluded from these studies (12–14), thus creating concerns regarding the safety and generalizability of outcomes for IBD individuals (15). Furthermore, patients with IBD on immune-modifying therapies have a higher risk of suboptimal vaccine response and unknown safety (16–18).

China began its COVID-19 vaccination campaign on December 19, 2020, for people at a higher risk of infection, such as the personnel responsible for the inspection and quarantine of the imported or exported cold-chain goods (products or materials that are required to be stored at a specific low temperature), the medical and health workers, and the transportation personnel. Since January 09, 2021, the campaign was extended to the general public unless they were in an active or uncontrolled stage of chronic disease, having acute disease, pregnant, allergic to vaccine components, and having other contraindications evaluated by experts (http://www.gov.cn). At the time of the survey, one type of inactivated viral vaccine was available for Chinese and four other vaccines (two inactivated vaccines, one recombinant protein vaccine, and one adenovirus vector vaccine) were in Phase III clinical trial. Internationally, IBD populations are suggested to use SARS-CoV-2 vaccines as soon as possible regardless of immune-modifying therapies (19, 20). However, in China, at present, there are no official recommendations about whether or not patients with IBD could take the vaccination. It is mainly based on their wish after seeking medical advice from their physicians or other authoritative resources. Therefore, we designed this questionnaire to investigate the attitudes and views of individuals with IBD on COVID-19 vaccines with the aim of achieving a better understanding of vaccine acceptance and/or hesitancy in this particular population.

## Materials and Methods

### Study Design, Setting, and Participants

This study was a questionnaire survey conducted among patients with IBD in China about their attitudes and views on COVID-19 vaccination. The questionnaire was reviewed by experts in IBD and pilot-tested in 10 patients with IBD to verify the readability and importance of content. The electronic questionnaire was created using Wen Juan Xing (an online survey tool by Changsha Ranxing Information Technology Co., Ltd., Hunan, China). It was then broadcasted on several WeChat (a social media application operated by Tencent Co., Ltd., Shenzhen, China) groups, where a subset of patients with IBD gathered from all over the country. Patients completed the questionnaire independently and voluntarily in the context of anonymity and free of charge. Each participant had only one chance to answer the questionnaire, and they could submit it only after responding to all the required questions, as prompted by the electronic system. Parents can assist their children or adolescents if they were unable to complete the survey independently. This study was approved by the institutional ethical review committee at the Second Affiliated Hospital of Nanjing Medical University ([2020]KY106).

### Questionnaire Design

A self-administered questionnaire was designed based on the literature review and clinical experience of the specialists in the management of patients with IBD during the COVID-19 epidemic. It consisted of 21 questions focusing on the views and attitudes of patients with IBD on COVID-19 vaccines. Besides demographic information of the participants, characteristics of IBD, current IBD treatments, and impact of COVID-19 pandemic on patients with IBD were also covered in the questionnaire as the potential factors influencing their attitudes toward vaccination. The choice of answers to the attitudes toward COVID-19 vaccination included “Yes,” “Not right away, but later,” “Undecided,” and “No.” A multiple-choice question was set for investigating reasons for COVID-19 vaccination. Participants who chose “Yes” or “No” were required to select the three main reasons for their respective choices. While those who chose “Not right away, but later” or “Undecided” had to respond to both the questions (see the questionnaire in Supplementary File 1).

### Statistical Analysis

Data collection and statistical analysis were carried out using the SPSS software system (SPSS for Windows, Version 23.0, IBM Corp, Armonk, United States). The electronic questionnaires were exported directly and were checked by two researchers independently to prevent vulnerabilities in the web system. Continuous variables were presented as median (interquartile range) for abnormal distribution and analyzed by Kruskal–Wallis H test. Categorical data were described as numbers (percentages) and tested by Chi-square analysis or Fisher's exact test. Cramer's V is an index that provides the correlation strength of classified variables (“0.1–0.3”: weak correlation, “0.3–0.5”: moderate correlation, and “≥0.5”: strong correlation). Pairwise comparison of Chi-square test was used to determine the differences between groups (*P* values were adjusted according to Bonferroni method), and Binary Logistic Regression was used for multivariate analysis of the screened factors. *P* < 0.05 and adjusted *p* < 0.05/(time of comparisons) were considered statistically significant for all the analyses.

## Results

From December 31, 2020, to January 3, 2021, a total of 543 electronic questionnaires were returned. We confirmed that all of them were complete, standardized, and qualified for analysis (543/543), with an effective rate of 100%. Our questionnaire covered 106 cities in 26 provinces of China. Areas with COVID-19 cases or new confirmed COVID-19 cases within 14 days were considered as a medium- to high-risk areas of COVID-19 based on the criteria set by the government (http://www.gov.cn). In our study, 63.2% of participants were from the area with medium-to-high COVID-19 risk in the past and/or at present. Less than one in six (15.3 %) patients or their family members had medical or biomedical backgrounds. Table 1 demonstrates the demographics of participants and their exposure to COVID-19.

**Table 1:** The demographics of participants and their exposure to COVID-19.

**Items**	**Results**
Total number	543
Gender, male, *n* (%)	343 (63.2)
Age, median (IQR)	35 (27–44)
Educational background, *n* (%)	
Junior High School or below	112 (20.6)
Senior High School degree	125 (23.0)
Associate degree	164 (30.2)
Bachelor's degree	114 (21.0)
Master's degree or above	28 (5.2)
Medical or biomedical background^*^, *n* (%)	
With	83 (15.3)
Without	460 (84.7)
Epidemic (medium-to-high) risk city of COVID-19 (past and/or present), *n* (%)	
Yes	343 (63.2)
No	200 (36.8)
Residential area, *n* (%)	
Cities	351 (64.6)
Villages	192 (35.4)
The people around you has a history of positive nucleic acid test for COVID-19, *n* (%)	
Yes	47 (8.7)
No	496 (91.3)
History of positive nucleic acid test for SARS-CoV-2	
Yes	6 (1.1)
No	537 (98.9)

*IQR, interquartile range*.

* *Medical or biomedical background: participant themselves or their friends/families are engaged in medical- or biomedical-related work*.

### Characteristics of Patients and IBD Medications

More than half of the respondents had Crohn's disease (69.8%) and the remainder had ulcerative colitis (25.2%) or indeterminate IBD (5.0%). There were 82.0% of participants in remission. 5-Aminosalicylates were the most commonly used medications (37.6%), followed by immunosuppressants (34.8%) and biological agents (33.0%). Patients with IBD who were taking biological agents, immunosuppressants, or corticosteroids (alone or in combination) for long-term were regarded as those in the immunocompromised status (67.0%). The demographics and characteristics of the patients are detailed in Table 2.

**Table 2:** Characteristics and medications for patients with IBD at present.

**Items**	**Results**
Disease category, *n* (%)	
UC	137 (25.2)
CD	379 (69.8)
Indeterminate IBD	27 (5.0)
Age at diagnosis, median (IQR)	29 (21-37)
Disease activity, *n* (%)	
Remission	445 (82.0)
Non-remission	98 (18.0)
History of intestinal resection, *n* (%)	
Yes	120 (22.1)
No	423 (77.9)
Current medical treatment for IBD [yes, *n* (%)]	
5-Aminosalicylates	204 (37.6)
Immunosuppressants	189 (34.8)
Biological agents	179 (33.0)
Fecal microbiota transplantation	93 (17.1)
Probiotics	65 (12.0)
Traditional Chinese medicine	46 (8.5)
Corticosteroids	20 (3.7)
Antibiotics	19 (3.5)
Nothing	24 (4.4)
The immunocompromised status, *n* (%)	
Yes	364 (67.0)
No	179 (33.97)

*UC, ulcerative colitis; CD, Crohn's disease; IBD, inflammatory bowel disease; IQR, interquartile range*.

### Attitudes and Views of Patients With IBD on SARS-CoV-2 Vaccination

**Figure 1:**
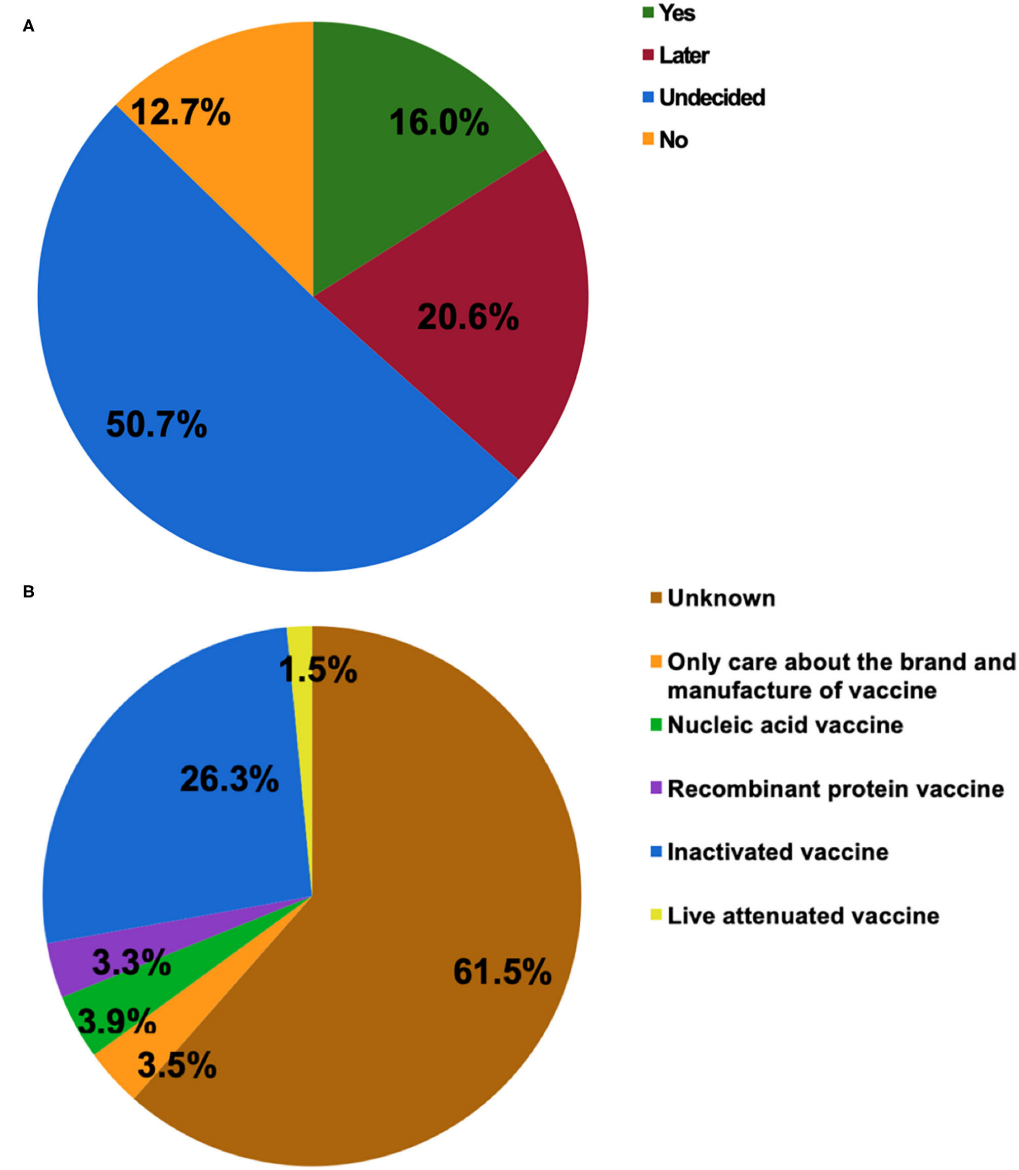
The attitudes of patients with inflammatory bowel disease (IBD) toward COVID-19 vaccination **(A)**. The type of COVID-19 vaccines that participants would prefer to receive **(B)**.

Figure 1A shows the attitudes of patients with IBD toward SARS-CoV-2 vaccination. Half of the participants were indecisive about vaccination (50.7%), which was consistent with the finding that 61.5% of them had no idea about what type of vaccine they would prefer to receive (Figure 1B). Those who had a clear attitude toward either being vaccinated or not accounted for 16.0 and 12.7%, respectively. The inactivated COVID-19 vaccine was the most popular of the four types of vaccines (26.3%). In the present study, most of the respondents agreed to SARS-CoV-2 vaccination as a vital measure for self-health (79.2%) or the health of others (85.6%).

Five factors (gender, medical or biomedical background, immunocompromised status, immunosuppressant usage, and the importance of vaccination for the health of others) were found to have a weak association with the attitude of individuals with IBD toward SARS-CoV-2 vaccination (Chi-square test, *p* < 0.035, Cramer's V = 0.181, 0.132, 0.126, 0.132, and 0.297 respectively). The importance of SARS-CoV-2 vaccination for self-health had a moderate relationship with the attitudes of participants toward vaccination (Chi-square test, *p* < 0.001, Cramer's V = 0.359). No other significant difference was found based on disease category, disease activity, treatment with fecal microbiota transplantation, and other parameters (Tables 1, 2).

**Figure 2:**
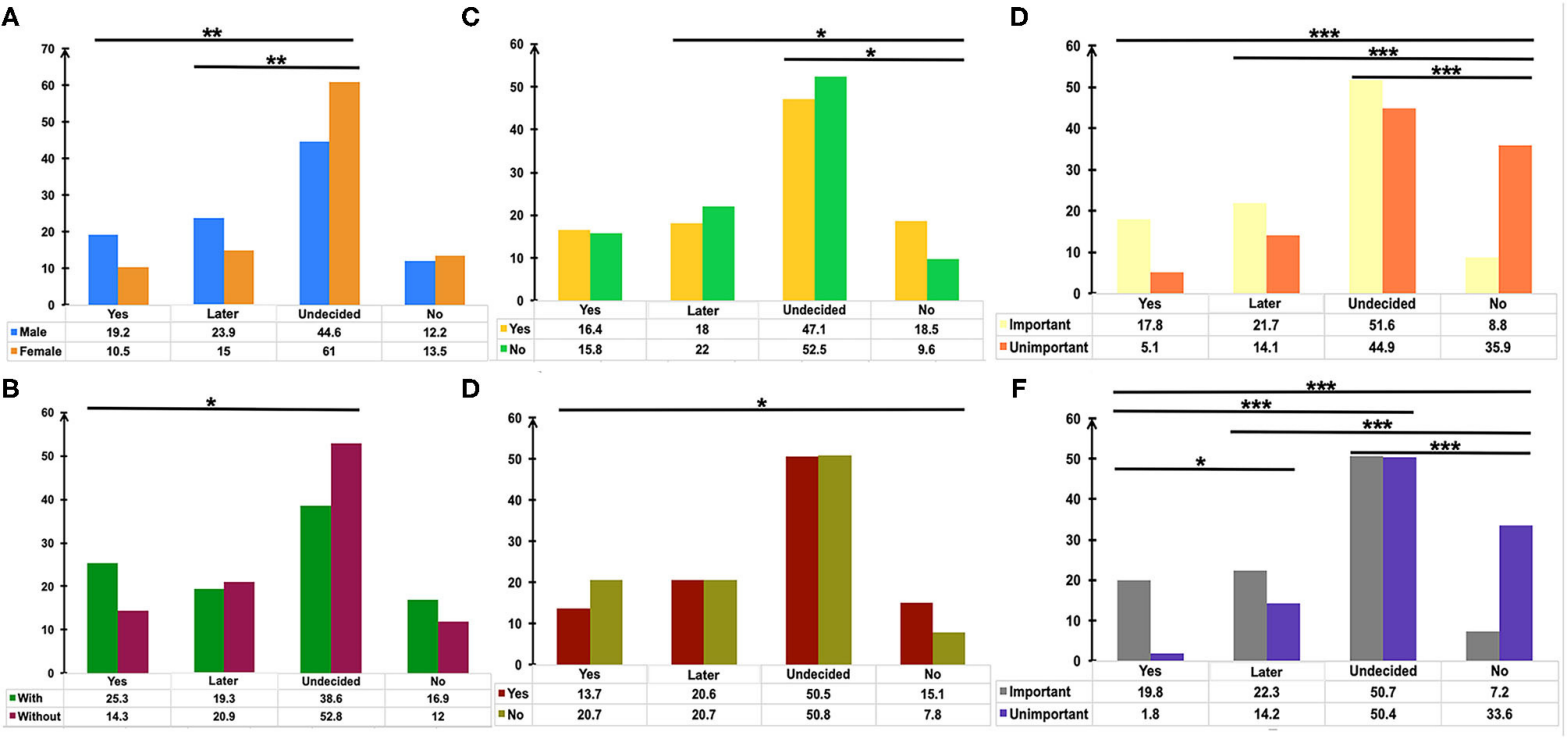
The potential factors influencing the attitudes of patients with IBD toward COVID-19 vaccination. Pairwise comparison of Chi-square test was used to determine the differences of gender **(A)**, medical or biomedical background **(B)**, immunosuppressants usage **(C)**, immunocompromised status **(D)**, the importance of vaccination for the health of others **(E)**, the importance of vaccination for self-health **(F)** between groups. *P* values were adjusted according to the Bonferroni method. *p < 0.0083, **p <0.0017, ***p < 0.00017.

Pairwise comparison of the Chi-square test was used to determine the differences between groups (Figure 2). The adjusted *p* < 0.0083 was considered statistically significant. Men showed a higher rate of choosing “Yes” or “Not right away, but later” in comparison with women who tended more toward choosing “Undecided” (Figure 2A, *p* = 0.001 and *p* = 0.001, respectively). Participants with medical or biomedical background had a higher rate of acceptance for the vaccination, while those without it were more likely to choose “Undecided” (Figure 2B, *p* = 0.004). Immunocompromised patients or patients on immunosuppressants tended toward not getting the vaccination (Figures 2C,D, *p* < 0.006). Overall, participants who considered vaccination critically important to self-health or the health of others chose to get vaccinated immediately or later, although 50.7% of patients were undecided about the SARS-CoV-2 vaccination. However, people who did not recognize the role of SARS-CoV-2 vaccines had a higher rate of not getting vaccinated (Figures 2C,D, *p* < 0.003). Table 3 shows the result of Binary Logistic Analysis between different groups.

**Table 3:** The independent factors impacting the attitude of patients toward SARS-CoV-2 vaccination, identified by Binary Logistic Analysis.

**Groups**	**Factors**	**Multivariate**	
	**Subgroups**	**OR (95% CI)^#^**	***P* value**
“Undecided” vs. “Yes”	Gender		0.002
	Female	2.43 (1.39–4.26)	
	Male	–	
	The importance of COVID-19 vaccination for self-health		0.001
	No	10.54 (2.50–44.42)	
	Yes	–	
	Medical or biomedical background^*^		0.009
	Without	2.37 (1.24–4.53)	
	With	–	
“No” vs. “Undecided”	Current immunosuppressant usage		0.023
	Yes	1.92 (1.09–3.39)	
	No	–	
	The importance of COVID-19 vaccination for self-health		0.005
	No	2.97 (1.39–6.34)	
	Yes	–	
“Yes” vs. “No”	The immunocompromised status		0.151
	Yes	0.53 (0.22–1.26)	
	No	–	
	The importance of COVID-19 vaccination for the health of others		0.248
	Yes	2.35 (0.55–10.03)	
	No	–	
	The importance of COVID-19 vaccination for self-health		<0.001
	Yes	31.17 (6.35–153.03)	
	No	–	
“Not right away, but later” vs. “No”	The importance of COVID-19 vaccination for the health of others		0.001
	Yes	4.73 (1.95–11.50)	
	No	–	
	Current immunosuppressant usage		0.134
	Yes	1.91 (0.69–5.27)	
	No	–	
	The importance of COVID-19 vaccination for self-health		<0.001
	Yes	31.17 (6.35–153.03)	
	No	–	

*OR, odds ratio; CI, confidence interval*.

* *Medical or biomedical background: Participants themselves or their friends/families are engaged in medical- or biomedical-related work*.

# *The OR value presents the comparison of vaccination intention (the former in the groups) in the subgroups, for example, in the groups of “Undecided” and “Yes,” women were 2.433 times more likely to choose “Undecided” than men (95% CI: 1.387–4.255, p = 0.002)*.

### Reasons Affecting Attitudes Toward SARS-CoV-2 Vaccination

**Figure 3:**
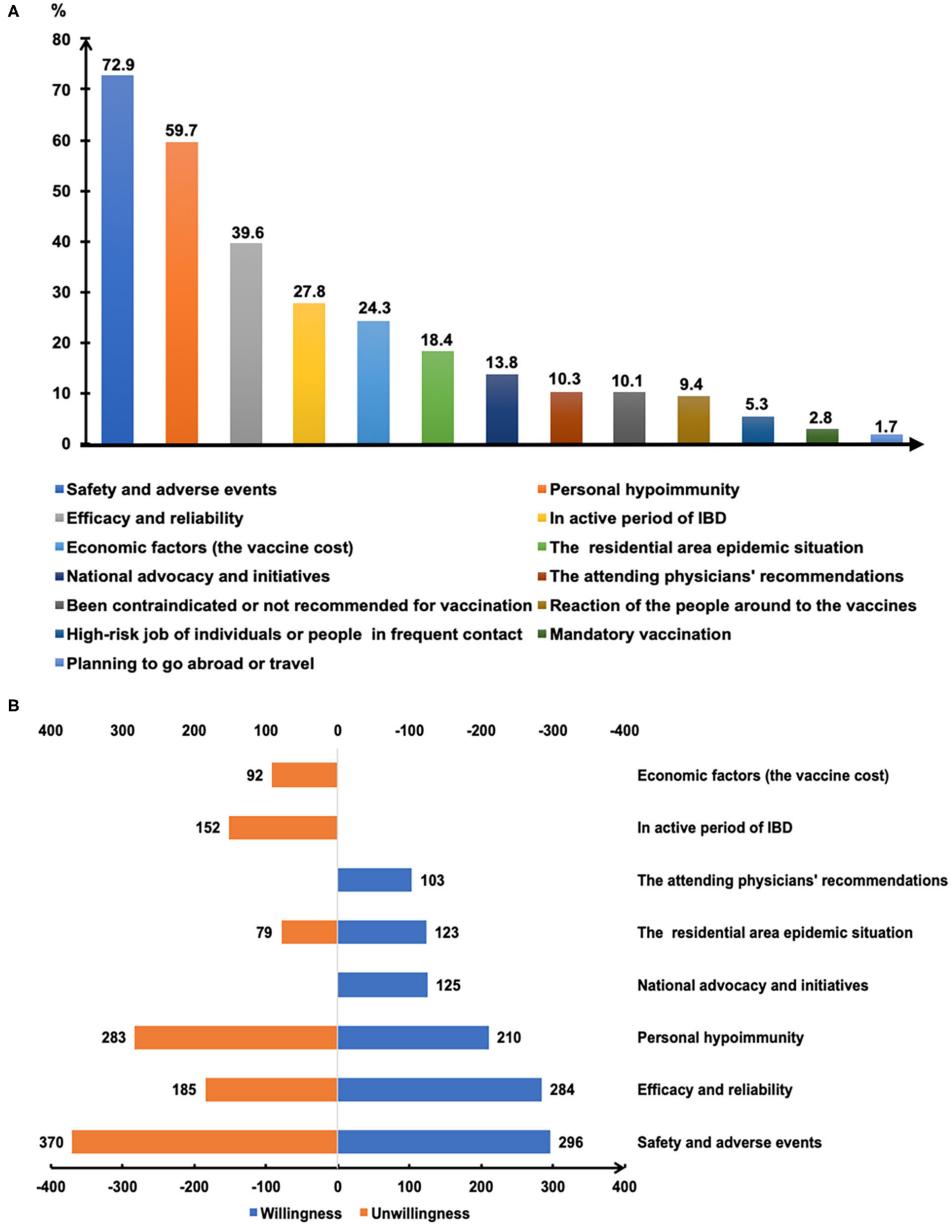
The reasons affecting COVID-19 vaccination of patients with IBD **(A)**. The main reasons for getting or not getting COVID-19 vaccinated **(B)**.

Figure 3 depicts the major reasons that influence the willingness for SARS-CoV-2 vaccination. Safety and adverse reactions, personal hypoimmunity, and efficacy and validity of COVID-19 vaccines were the top three concerns of patients that were independent of their willingness toward vaccination (Figure 3B). Notably, the national initiatives and recommendations from attending physicians were also the main reasons for some patients getting vaccinated.

## Discussion

The COVID-19 pandemic has brought great trouble and concerns for patients with IBD. In our present survey, 63.2% of patients came from the COVID-19 risk area and 1.1% of those patients had a positive history of SARS-CoV-2 infection (Table 1). Safe and effective vaccines are expected by health authorities and the medical community as the most powerful weapon against the COVID-19 pandemic (21), and nearly 80.0% of respondents in our study also recognized that SARS-CoV-2 vaccination was critical to the self-health and health of others.

Willingness to be vaccinated matters greatly, for the reason that a sufficient vaccination rate in a population is indispensable before herd immunity is achieved. The published expert consensus and opinions recommend vaccinating all IBD populations as soon as they are able to receive SARS-CoV-2 vaccines (15, 19, 20). In the present study, COVID-19 vaccination hesitancy existed in 50.7% of patients with IBD and merely 16.0% of participants opted for vaccination (Figure 1). The attitude of the participants towards the COVID-19 vaccine was significantly more negative than that of the general population in China and the patients with IBD from other countries (22–24). It was reported that 67.1% of the general population in China were willing to accept the COVID-19 vaccination during the same period (22). Furthermore, the rate of vaccination intent among IBD participants in our present study was lower than that among the IBD population in Boston (80.9%) (23) and Italy (80.3%) (24). Similarly, a lower rate of intent for hepatitis B (18.8%) and varicella vaccines (6.6%) was observed among patients with IBD in China (25). The potential reasons could be the insufficient knowledge about vaccination and many concerns about vaccine safety in this specific population, as reflected in our study and other reports (16–18, 23).

Gender was found to be one of the determinants of attitude towards COVID-19 vaccination in this study (Figure 2A), in agreement with several other surveys (26, 27). The reasons for vaccination hesitancy in the female group needs to be further explored in the future, for concerns regarding reproductive impact, personality trait, and others. Patients with IBD with medical or biomedical background had a higher rate of immediately getting vaccinated (Figure 2B), and 21.7% of the respondents considered the recommendations of their attending physicians as one of the three main reasons for getting vaccinated (Figure 3B). An anonymous cross-sectional survey conducted among Chinese adults reported that the recommendation of the doctor was a contributing factor in the decision-making for vaccination (27). As recommended by clinical practice guidelines for vaccination of the immunosuppressed host (28), IBD specialists should share the responsibility of communicating with their patients about the recommendations and appropriate vaccinations amidst the COVID-19 pandemic.

Most vaccines are broadly recommended for patients with IBD, including the COVID-19 vaccines (19). However, the vaccine hesitancy and unwillingness are particularly pronounced among patients with IBD mainly due to the unpredictable outcome amidst disease activity and immunocompromised status, together with the blunted immunogenicity of some vaccines found in the immune-modifying therapies (16–18, 23, 29). Similarly, we demonstrated that the acceptance of COVID-19 vaccination was lower in participants being treated with immune-modifying therapies, especially in those using immunosuppressants (Figures 2C,D). Moreover, previous clinical studies of COVID-19 vaccines have excluded patients with immune conditions (12–14), thus heightening the concerns of patients with IBD about the safety and generalizability of the outcomes (15). We found that safety and adverse events, personal hypoimmunity, as well as efficacy and validity of COVID-19 vaccines are the top three concerns of patients with IBD for COVID-19 vaccination (Figure 3B). This phenomenon was commonly shared in the IBD populations from other countries (23). Therefore, the expert panel suggested that patients with IBD receiving SARS-CoV-2 vaccination should be referred to prospective registries tracking the amplitude and duration of immune responses across different vaccine platforms (19).

Although half of the patients with IBD were undecided about SARS-CoV-2 vaccination, participants who realized the vital importance of vaccination to self-health or the health of others had a higher intention for immediate or later vaccination when compared to those who did not fully appreciate its importance (Figures 2E,F). This result highlights the role of health education (23, 25) and recommendations from authoritative sources, such as government and IBD specialists, in removing some barriers along the process of vaccination (Figure 3B).

The vaccine candidates can be grouped into protein subunit vaccines, nucleic acid vaccines, whole-inactivated viral vaccines, live attenuated viral vaccines, and others (30). In China, more than 177.7 million doses of vaccines have been administrated to the general population (accessed August 6, 2021) (1). In general, the patients with IBD can safely receive the inactivated vaccines for vaccine-preventable diseases irrespective of immune conditions (19, 29), which may be the reason that the inactivated COVID-19 vaccine was the most popular of the four types of vaccines (Figure 1B). In reality, the types of SARS-CoV-2 vaccines including the inactivated vaccine, messenger RNA vaccines, replication-incompetent vector vaccines, and recombinant vaccines are considered safe for patients with IBD (19). The BNT162b2, ChAdOx1 nCoV-19, and mRNA-1273 vaccines have received regulatory approval in the United Kingdom, which applies to patients with IBD, indicating that an immunosuppressed state is not a contraindication (20). Even so, more research should be carried out highlighting the safety and efficacy of COVID-19 vaccines in this particular population.

This study has some limitations. First, we conducted the survey *via* an electronic questionnaire. Thus, although the disadvantages of the paper questionnaire, such as skipped questions or the time-consuming task of uploading the collected data, were avoided, the data of patients who could not operate electronic devices were lost. Second, response bias inherent to the online survey and an abbreviated study period may have led to an inaccurate understanding of vaccination intent. Finally, our survey results did not completely represent the attitude or the concerns of IBD populations on COVID-19 vaccination from other countries, due to different situations of the pandemic globally.

In conclusion, this study discloses COVID-19 vaccination hesitancy and paucity of knowledge about the COVID-19 vaccines in the IBD population. Further studies evaluating the efficacy and safety of various COVID-19 vaccines in IBD individuals, with a specific focus on the impact of immune-modifying therapies and serologic responses, should be carried out to alleviate the concerns of such patients. Health education and recommendation from authoritative sources, such as government and IBD specialists, may facilitate COVID-19 vaccination efforts.

## Data Availability Statement

The original contributions presented in the study are included in the article/Supplementary Material, further inquiries can be directed to the corresponding author/s.

## Ethics Statement

This study was approved by the Second Affiliated Hospital of Nanjing Medical University institutional ethical review board ([2020]KY106).

## Author Contributions

FZ, BC, GJ, XW, and JL designed the study. XW did the statistical analysis. XW and JL drafted the manuscript. HB edited the manuscript for professional English. QD, BC, and GJ were involved in the revision of the manuscript. All authors have read and approved the final version of this manuscript.

## Funding

This study was supported by the publicly donated Intestine Initiative Foundation; the National Natural Science Foundation of China (81600417); and the 789 Outstanding Talent Program of SAHNMU (789ZYR20200802250).

## Conflict of Interest

The authors declare that the research was conducted in the absence of any commercial or financial relationships that could be construed as a potential conflict of interest.

## Publisher's Note

All claims expressed in this article are solely those of the authors and do not necessarily represent those of their affiliated organizations, or those of the publisher, the editors and the reviewers. Any product that may be evaluated in this article, or claim that may be made by its manufacturer, is not guaranteed or endorsed by the publisher.
