# Supplementary material for: The COVID-19 Vaccination Hesitancy Among the People With Inflammatory Bowel Disease in China: A Questionnaire Study
Source: Front Public Health. 2021 Oct 11;9:731578. doi: 10.3389/fpubh.2021.731578 (PMC8542757; doi:10.3389/fpubh.2021.731578)
Supplement: Supplementary file 2 [file Table_2.DOCX]

**The attitude and view of patients with inflammatory bowel disease (IBD) to COVID-19 vaccination**

Dear participants:

We sincerely invite you to participate in the questionnaire survey about “The attitude and view of patients with inflammatory bowel disease (IBD) to COVID-19 vaccination”. There are 21 questions in total, which will take 3-5 minutes. Your answer will be kept confidential! There are no standard answers to the questionnaire. Please identify the corresponding options. You need to complete the questionnaire independently. Thank you!

**Part one: The basic information**

1. What's your IBD diagnosis?
   1. CD
   2. UC
   3. Indeterminate IBD
2. Disease activity of you
   1. Remission
   2. Active
3. Where are you from? _________(Province) _________(City)
4. Where do you live?
   1. Cities
   2. Villages
5. What is your gender?
   1. Male
   2. Female
6. What is your current age? _________(Year)
7. What was your approximate age of diagnosis? _________(Year)
8. What is your educational background?
   1. Junior high school or below
   2. Senior high school degree
   3. Associate degree
   4. Bachelor’s degree
   5. Master’s degree
   6. Doctor’s degree
9. Do you/your relatives have the background of professional medical/biomedical knowledge?
   1. Yes
   2. No
10. Have you ever been tested positive for COVID-19?
    1. Yes
    2. No
11. What is/are your current IBD treatment(s)? (Multiple-Choice question)
    1. Infliximab (Remicade)
    2. Adalimumab
    3. Certolizumab (Cimzia）
    4. Vedolizumab（Entyvio）
    5. Ustekinumab（Stelara）
    6. Azathioprine（Imuran），6-MP or methotrexate
    7. Mesalamine or sulfasalazine therapies
    8. Tofacitinib
    9. Thalidomide
    10. Tacrolimus
    11. Sirolimus
    12. Cellcept
    13. Antibiotics（e.g. ciprofloxacin, flagyl or vancomycin）
    14. Ciclosporin
    15. Steroids
    16. FMT (Fecal microbiota Transplantation)
    17. Probiotics
    18. Traditional Chinese medicine
    19. None
12. Do you have a history of intestinal resection for IBD?
    1. Yes
    2. No
13. Do you have a history of influenza vaccination during October to December, 2020?
    1. Yes
    2. No
14. Someone close to me was negatively affected by COVID-19.
    1. Yes
    2. No

**Part two: Attitudes and views of IBD patients toward SARS-CoV-2 vaccination**

1. I think the COVID-19 vaccination is critical to the health of others.

1. Yes
2. No

2. I think the COVID-19 vaccination is important for my health.

1. Yes
2. No

3. Reasons that affect my vaccination. (Multiple-Choice question)

- 1. Efficacy and reliability of vaccine
  2. Safety and adverse events of vaccine
  3. National advocacy and initiatives
  4. The residential area epidemic situation
  5. In active period of IBD
  6. Personal hypoimmunity
  7. Economic factors (e.g. the vaccine cost)
  8. The attending physician's recommendations
  9. Reaction of the people around to the vaccine
  10. Planning to go abroad or travel
  11. High-risk job of individuals or people in frequent contact
  12. Been contraindicated or not recommended for vaccination
  13. Mandatory vaccination

1. Do you plan to receive the COVID-19 vaccines？
   1. Yes
   2. Later in the year
   3. Undecided
   4. No
2. Which are the three main reasons that you are willing to receive the vaccine? (Who choose “Yes”, “Later in the year” or “Undecided”)
   1. Efficacy and validity of vaccine
   2. Safety and adverse events of vaccine
   3. National advocacy and initiatives
   4. The residential area epidemic situation
   5. In active period of IBD
   6. Personal hypoimmunity
   7. Economic factors (the vaccine cost)
   8. The attending physician's recommendations
   9. Reaction of the people around to the vaccine
   10. Planning to go abroad or travel
   11. High-risk job of individuals or people in frequent contact
   12. Been contraindicated or not recommended for vaccination
   13. Mandatory vaccination
3. Which are the three main reasons that you are unwilling to receive the vaccine? (Who choose “Later in the year”, “Undecided” and “No”)
   1. Efficacy and validity of vaccine
   2. Safety and adverse events of vaccine
   3. National advocacy and initiatives
   4. The residential area epidemic situation
   5. In active period of IBD
   6. Personal hypoimmunity
   7. Economic factors (the vaccine cost)
   8. The attending physician's recommendations
   9. Reaction of the people around to the vaccine
   10. Planning to go abroad or travel
   11. High-risk job of individuals or people in frequent contact
   12. Been contraindicated or not recommended for vaccination
   13. Mandatory vaccination
4. Which one of the COVID-19 vaccines do you prefer to receive?
   1. Viral inactivation vaccine
   2. Recombinant protein vaccine
   3. Adenovirus vector vaccine
   4. Nucleic acid vaccine
   5. Only care about the brand and manufacture of vaccine
   6. Have no idea of it

The questionnaire has been completed. Thank you for cooperation!
